# A dataset without a code book: ethnography and open science

**DOI:** 10.3389/fsoc.2024.1308029

**Published:** 2024-03-01

**Authors:** Shamus Khan, Jennifer S. Hirsch, Ohad Zeltzer-Zubida

**Affiliations:** ^1^Departments of Sociology and American Studies, Princeton University, Princeton, NJ, United States; ^2^Mailman School of Public Health, Columbia University, New York City, NY, United States; ^3^Department of Sociology, Princeton University, Princeton, NJ, United States

**Keywords:** ethnography, open science, epistemology, qualitative method, methodology

## Abstract

This paper reflects upon calls for “open data” in ethnography, drawing on our experiences doing research on sexual violence. The core claim of this paper is not that open data is undesirable; it is that there is a lot we must know before we presume its benefits apply to ethnographic research. The epistemic and ontological foundation of open data is grounded in a logic that is not always consistent with that of ethnographic practice. We begin by identifying three logics of open data—epistemic, political-economic, and regulatory—which each address a perceived problem with knowledge production and point to open science as the solution. We then evaluate these logics in the context of the practice of ethnographic research. Claims that open data would improve data quality are, in our assessment, potentially reversed: in our own ethnographic work, open data practices would likely have compromised our data quality. And protecting subject identities would have meant creating accessible data that would not allow for replication. For ethnographic work, open data would be like having the data set without the codebook. Before we adopt open data to improve the quality of science, we need to answer a series of questions about what open data does to data quality. Rather than blindly make a normative commitment to a principle, we need empirical work on the impact of such practices – work which must be done with respect to the different epistemic cultures’ modes of inquiry. Ethnographers, as well as the institutions that fund and regulate ethnographic research, should only embrace open data after the subject has been researched and evaluated within our own epistemic community.

## Introduction

Open science has reached ethnography. Yet the problems open science identifies and the solutions open science proposes are grounded in the epistemic logic of particular (i.e., non-ethnographic) scholarly communities. Importing these problems and solutions to other epistemic communities requires consideration of what might be lost in translation. Before ethnographers embrace open science, we must ask, “do the asserted benefits of open science—improved data quality, data reliability, and increased trust in the scholarly enterprise—hold true for ethnographic research?” These normative-laden claims are, as of yet, entirely empirically unsubstantiated within ethnographic practice. In fact, the evidential base for the open science movement in general is relatively weak. Moreover, certain epistemic foundations of ethnographic research—positionality and normativity—are consequential for but not reckoned with in the open science movement.

In this paper we take on one small part of the open science movement: the question of data availability, or what we will call “open data.” Drawing on the work of Karin Knorr Centina, we argue that as we consider importing open data practices to ethnography, we need recognize ethnography’s “epistemic culture.” Knorr Centina defines epistemic cultures as “those amalgams of arrangements and mechanisms—bonded through affinity, necessity, and historical coincidence—which, in a given field, make up *how we know what we know*. Epistemic cultures are cultures that create and warrant knowledge” (1999:1). Whereas some view science as “universal,” (which Knorr Centina calls the “monist” position), with a singular approach to methods, logic of inquiry, a shared understanding of reasoning, and a specific approach to the relationship between theory and data, the idea of “epistemic cultures” suggests that different fields of scientific inquiry can vary quite considerably in how they understand the logic of science. Knorr Centina developed her argument with respect to two natural science fields: high energy physics and molecular biology. In this paper we extend her insights to the social sciences, considering how the epistemic culture of ethnography—which is to say how it thinks about method, logic of inquiry, reason, and the relationship between theory and data—is distinct from other epistemic cultures. This different does not necessarily make ethnography “less scientific” but instead, “differently scientific.”

One could think of the open data movement as a colonizing force, subsuming the cultural logics of different scientific communities under its normative demands, demands which are grounded in the presumption of a singular, or monist scientific approach. In bringing “backward communities” into the light, it promises that the quality and respectability of knowledge will increase, and so too will its power. Yet the disunity of scientific communities—as Knorr-Centina describes—means that such a universalizing impulse is likely both to be met with resistance from those whose primary method is ethnography and to yield distinct outcomes within different epistemic cultures. Not only may some open science practices (and their intended outcomes) get lost in translation, but some epistemic communities may also find themselves deemed “illegitimate” should they fail to comply with a set of external demands. These concerns are recognized by advocates of the broader open science movement. In their review of replication in social science, Jeremy Freese and David Paterson note that open science practices are likely to be adopted in different ways within different epistemic cultures. “The role of replication within a field ought to be understood as the outcome of a process of cultural development which is influenced by both internal dynamics and external pressures rather than a universal feature of an idealized scientific method” (2017: 151). The external pressures they speak of, however, also suggest that for a form of inquiry to be considered a “science” it is increasingly conditional on its embrace of open science principles. Those powerful epistemic cultures that have embraced and constituted the logic of open science are likely to suggest that reluctance of other communities to conform to their favored logic implies they are not truly “scientific.” Such work to determine legitimacy of knowledge has long been recognized as a kind of power politics. The colonizing advance is one that places certain epistemic approaches above others in their legitimacy.

Describing the epistemic culture of ethnography is not without its own perils. Ethnographers reside in a methodologically contentious corner of sociology; indeed, in recent years perhaps the most vigorous and impassioned recent debates within the field of sociology have been among ethnographers (see Duneier, 2002, 2006; Wacquant, 2002; Klinenberg, 2006; Jerolmack and Khan, 2014a,b). Even defining the ethnographic community itself is a challenge. We conceptualize ethnography—which includes both participant observation and in-depth interviews—as a relational-interactive method. A necessary condition for data gathering is that a researcher personally interacts with and enters a relationship with the research subjects (this can include digital ethnographies, where interactions happen within online spaces). Because of these interactive and relational components, the ethnographic community tends to rest upon two epistemic foundations: reflexivity and positionality. Positionality “reflects the position that the researcher has chosen to adopt within a given research study” (Savin-Baden and Major, 2023: 71). It also encompasses the position that is relationally constructed by research subjects themselves, and grounded in the attributes of the researcher (e.g., race, gender, sexuality, class, ability, etc.). The concept of positionality is, in part, grounded in the Black feminist perspective which argues that who we are and how we interact with the world shapes what we find (Collins, 1986, 1999, 2000; see also the perspective in Smith, 1989). Synthesizing this and other insights, Homes argues (Holmes, 2020),

The term positionality both describes an individual’s world view and the position they adopt about a research task and its social and political context. The individual’s world view or ‘where the researcher is coming from’ concerns ontological assumptions (an individual’s beliefs about the nature of social reality and what is knowable about the world), epistemological assumptions (an individual’s beliefs about the nature of knowledge) and assumptions about human nature and agency (individual’s assumptions about the way we interact with our environment and relate to it) (2020: 1).

While such positionality is not unique to ethnographic work, it is more acute because of the relational-interactive necessity of gathering data in interview and participant observation contexts. This reflects an epistemic culture wherein Haraway’s (1991) classic arguments about the inherently situated nature of knowledge construction (and the inevitability of a partial perspective) are more fully embraced than they are in other scientific communities. From this perspective the question is not about how to minimize the impacts of positionality in order to guide us closer to objective observation, but instead is about how to establish “an agenda for the assessment of subjectivity” (Malterud, 2001: 484). Reflexivity is a central part of the agenda. As Malterud continues,

Reflexivity starts by identifying preconceptions brought into the project by the researcher, representing previous personal and professional experiences, pre-study beliefs about how things are and what is to be investigated, motivation and qualifications for exploration of the field, and perspectives and theoretical foundations related to education and interests. (2001: 484)

Such a reflexive epistemic approach starkly contrasts that of the more positivist open data movement. And it is of particular importance because successful replication (a core demand of the open science movement) requires the capacity to fully convey the observer’s position in the field and subjectivity as a person. That prospect is necessarily incomplete, thereby undermining the capacity of certain forms of replication.

We do not oppose the application of some open data principles to ethnographic research,^1^ but we do provide some words of caution based on our own research experiences. We suggest that open science principles should not be directly *adopted* by any epistemic community; instead, we suggest they may be *adapted* to recognize and reflect diverse epistemic cultures. Such adaptation requires empirical study in addition to good arguments, and empirical study has been curiously absent from those who advocate for open science principles. We argue, for example, that making our own research data broadly accessible would have interfered with our data gathering, lowered the quality of our data, and decreased the reliability of our findings. Yet other forms of data verification (e.g., hiring someone to independently check the claims in our book (Hirsch and Khan, 2020) against our fieldnotes and interview transcripts) likely increased readers’ trust in the reliability of our findings and arguments.

Our argument proceeds in three parts. First, we review the literature on open data, outlining the problems with scientific knowledge production and dissemination that open science purports to address. We also summarize the subsequent proposed solutions to these problems. We construct three logics that scholars have converged upon: epistemological, political-economic, and regulatory. We recognize the value of these arguments but note that they have not been grounded in the ethnographic enterprise nor do they recognize the epistemic culture of the ethnographic community, nor have they been rigorously studied. As might be anticipated, we highlight the first diagnosis—epistemic—as the most acute. But we also note how the distinct culture of ethnographic scientific production creates challenges for the political-economic and regulatory justifications for open data.

Second, we use examples from our own ethnographic research on sexual violence (Hirsch and Khan, 2020) to provide grounding and texture as we evaluate the implications of open data for ethnographers. We conclude by reflecting on the next steps we think the scholarly community should take to address the concerns we outline. Specifically, we argue for the importance of empirically evaluating the impact of open data and considering its implications for the specific epistemic culture of ethnographic work.

### Three justifications for open data in sciences

Open science is a broad movement that considers all aspects of the process of scientific production. This includes, but is not limited to, access to how data are generated (i.e., availability of research instruments), access to raw data, transparency of coding decisions/analysis and material/replication packages, and public access to scholarly outputs. In this paper we primarily focus on the second in that list of elements of the open science movement: what we refer to as “open data.” Open data focuses on the availability of the various empirical materials produced during research: the “raw data” that will subsequently be analyzed. We are highly supportive of other parts of the open science movement, such as the availability of research instruments, papers, and coding/analysis schemas.

We provide an abbreviated account of the literature on open data. We do not limit our focus to qualitative research or even social scientific research. Instead, we look at how a specific set of “problems of knowledge creation” are diagnosed across a range of disciplines. Scholars are not so naïve to propose open data as the *sole* solution to the problems they outline. We do not review the broader range of suggestions, beyond open data, in this paper. We also do not interrogate what scholars describe as the problem of knowledge creation and dissemination in-and-of themselves. We would note, however, that in our assessment, all the problems that scholars have identified are “real” and it is reasonable to be concerned about them. We would even agree that open data is, for many of them, a reasonable step toward a solution. Our argument is that the diversity of epistemic cultures and scientific communities warrants problem-framings and solutions that are sensitive to these contexts; open data faces challenges when uniformly applied. We also believe that the open science movement must encompass empirical work to evaluating its own claims—something that is curiously underdeveloped from such a “scientific” movement. Arguments for open data give three principal justifications: *epistemological, political-economic,* and *regulatory*. Table 1 provides a summary of the problems the open science movement has identified, these three justifications for the open science movement, and the proposed logic of the solution.

**Table 1:** The problems of knowledge production and dissemination and the logic of the open data solution.

Problem	Justification for open science	Logic of solution
*Reliability* Scholars make a range of decisions about how they gather and analyze data: how concepts are operationalized, what observations are categorized, what counterevidence is mobilized or ignored, etc. These decisions are often “black boxed,” thereby undermining reliability of data analysis.	*Epistemological*	Empirical observations are independently and uniquely re-observed, operationalization is systemically evaluated, and negative cases are re-explored to establish that measures and variables/relationships are reliably constructed. Claims are thereby re-established, and findings can be replicated. Alternatively, and typically, the same data is re-analyzed.
*Equity & Efficiency* Data is enormously expensive to collect, and it would be highly inequitable and undesirable to allow data to remain solely in the possession of those who could afford to collect it (and gain advantage from privileged access). The public often pays for research through government programs, but often does not have access to the information it funds.	*Political-economic*	Making data widely available addresses inequity by giving more people to access information. Various scholars working off the same data allows for a diversity of perspectives, wherein scholars can make inquiries that others may not have considered or may not have had the time to undertake. In addition, there is a normative claim that publicly funded data should be publicly available.
*Fraud* The stakes for scholars are extremely high. There are strong incentives to fabricate work to advance one’s career an argument the scholar has an interest in. Discovering fraud is extremely difficult.	*Regulatory*	Data accessibility increases the likelihood of discovery, thus discouraging fraud.

The first problem scholars identify is about *reliability*. Within certain epistemic cultures the ideal form of knowledge production is one where observations are independently and uniquely re-observed to establish that a measure is reliably constructed, the techniques of observation are accurately described and executed, the analysis is reproduceable, and findings can be replicated. In practice, this is often impractical – and sometimes impossible. Rare episodes can be enormously difficulty to re-observe; when dealing with human subjects, temporal constraints can make re-observation impossible. This suggests two kinds of replication: that of the study, and that of the analysis. In a review of the state of replication norms in sociology, Freese and Peterson offer a two-by-two matrix of replication practices in quantitative social science. The axes they identify are *similar/different*, and *old data/new data* (2017). They designate the two practices that work with existing data *Verifiability* (similar), direct reproduction of results with the same data and code, and *Robustness* (different), reexamination of the same data under different specifications and analyses. While the *new data* axis is the gold standard (i.e., testing for *Repeatability* (similar) or *Generalizability* (different)), openly accessible existing data provides the next best solution to empirical reliability.

It is also the form most likely to be used. In an editorial for *PLOS Computational Biology* laying out best practices for data-sharing and management, Goodman et al. (2014) note that “the amount of real data and data description in modern publications is almost never sufficient to repeat or even statistically verify a study being presented.” When data are presented to their audience, the full picture is rarely sufficient for critical examination of the findings. Operationalization decisions are blacked boxed. Potential negative cases are not all systematically presented. Making data accessible allows for the replication, reproduction, and validation of published results. Scholars can evaluate how raw data are operationalized into values. Cases that “do not fit” can more clearly be seen and evaluated by a community of scholars. Nosek et al. (2015) make the case in *Science* for journals to adopt author guidelines commensurate with open science ideals, arguing that “to progress, science needs both innovation and self-correction; replication offers opportunities for self-correction to more efficiently identify promising research directions.” This provides an epistemic justification for open-science principles.

The second problem scholars identify within the scientific community is about *equity and efficiency*. Across the natural and human sciences data can be enormously expensive to collect. This concern may have increased as “big data,” or omnibus data, have garnered more and more symbolic value. In response to such expense, scholars have expressed concern about equity. If data remains solely in the possession of those who can afford to collect it, then scholarly inequalities may be aggravated as those few scholars who can secure funding for large-scale data projects would be able to extract considerable rents from their privileged access. In addition to threats to equity, there is concern that knowledge itself might suffer as limiting who can access large data sets will narrow insights, either because it would constrain which problems are considered to be of interest or because having just one person work on a dataset would severely hinder the amount of work that could be done on those data. Finally, and perhaps most powerfully, given that research funding often comes at taxpayer expense, the public, broadly conceived, should have access to the data they paid for.

This second justification for open data, *political-economic,* is rooted in a normative commitment to the democratization of knowledge, and in a parallel commitment to efficiency in academic research. Efficiency arguments have two basic elements: the effective utilization of public funding and the increased potential for innovation due to cooperation and knowledge-sharing. A salient example of the latter arose in the wake of breakthroughs in human genome sequencing when there were consequent efforts to commodify such knowledge (Borgman, 2017, p. 208). A report published in *Science* as this debate was unfolding does well to demonstrate commitment to knowledge-sharing. Genomic experts who attended a workshop on the subject had responses ranging from cautious apprehension to outright alarm: “‘Being able to copyright the genome would make me very uncomfortable’, said Frank Ruddle of Yale. And Caskey asked if there is a precedent for saying, ‘This information is so important that it cannot be proprietary. This is the first time we’ll ever get this information on man—can we make a special case’?” (Roberts, 1987).

An argument for the effective use of public funding can be found the earliest framing of open data and its advantages (Borgman, 2017, p. 44). In their paper, Murray-Rust and Rzepa (2004) contend that “Most publicly funded scientific information is never fully published and decays rapidly.” Wilkinson et al. (2016) point to structural conditions, arguing that “Unfortunately, the existing digital ecosystem surrounding scholarly data publication prevents us from extracting maximum benefit from our research investments.” Similarly, there are those who highlight slower innovation in the absence of data sharing. Work studying researchers’ attitudes towards data sharing argues that “Recent studies have estimated the annual financial cost of not sharing FAIR [Findable, Accessible, Interoperable, and Reusable] data to be at least €10.2bn for the European economy, while the impact of FAIR on potential economic annual growth is estimated to be €16bn annually” (Pujol Priego et al., 2022).

The third justification for open data, *regulation*, asserts that transparent data practices can help deter academic fraud by increasing the likelihood of discovery. As the stakes of academic careers have increased beneath the challenges of contingent employment, more soft money positions, and the exacerbation of rewards to success/risks of failure, so have the temptations to artificially achieve success through forms of deception. Open data is seen as a preventative and proactive mechanism to limit academic fraud.

The authors of an early report about data-sharing, commissioned by the National Research Council, maintain that “The expectation that further analyses and comparisons will be conducted should discourage dishonest manipulations” (Fienberg et al., 1985). The characterization of these manipulations range from widespread bad practice—“researchers have incentives to analyze and present data to make them more ‘publishable,’ even at the expense of accuracy” (Miguel et al., 2014)—to malicious falsification—“One of the worst frustrations of scientists and decision makers is caused by a revelation or strong suspicion that information that was presumed correct and on which results, recommendations, or decisions were based is faulty. Reactions are particularly bitter when willful fabrication, falsification, or distortion of data is involved” (Fienberg et al., 1985).

Freese and Peterson argue that under a publication regime that expects authors to comply with open data standards, even cases that argue for an exemption will benefit from increased credibility (2017). In other words, even when a strong argument against the public availability of certain data can be made, an explicit disclaimer regarding the possibility that the data “can be verified independently in principle” could bolster the work’s reliability. Open data, by this logic, can be part of a regulatory apparatus that disincentivizes fraud through the increased likelihood that it could be discovered. In the context where the structure of academic careers has incentivized the artificial production of scholarly success, access to the raw material of scholarly inquiry could moderate harmful fraudulent behavior.

### Ethnography: what (if anything) gets lost in translation

The call for open science emerged within the natural sciences and has slowly made its way to the more quantitative social sciences. Those in disciplines with diverse methodological approaches (sociology, in particular) and epistemic cultures are thereby some of the first spaces to undertake the translational work of migrating open science into ethnographic practice. We say “translate” because different scientific approaches use different lexicons—drawing upon different logics of inference, different approaches to data gathering, documentation, and analysis, and different understandings of positionality and epistemology.

This translation raises several questions: (1) are reliability, equity & efficiency, and fraud problems in ethnography? (2) if so, are they similar in nature to the problems identified in the open data framework? (3) does the open data framework offer reasonable solutions to these problems? In this section we attempt to answer these questions for ethnographic work in the abstract by drawing upon a concrete reflection on our (Hirsch and Khan, 2020) own work on sexual violence. We find degrees of both congruence and divergence, suggesting significant insights are lost in translation if migrated to the field in too direct of a manner. Overall, we suggest that the problems identified are real, but the solution of open data may not solve these problems, and in some instances, may in fact damage data quality as well as the overall trust in ethnographic practice.

#### Reliability and replication

At its core, reliability is about whether findings can be replicated. This seems a simple enough concept. Either a finding can be reproduced, or it cannot. Quantitative replication is grounded in generating the same outcomes, broadly presented in two pathways: re-analyzing the same data to generate the same answer or drawing an equivalent sample to generate the same results. For ethnographers, given the unique situatedness of ethnographic knowledge construction, replication through these pathways presents significant challenges.

In the first pathway, if Scholar A generates an association between two variables, then Scholar B should be able to as well, by following Scholar A’s techniques with Scholar A’s data. Yet ethnographic fieldwork is unlikely to be used in this way. First, because fieldnotes do not contain all the information a scholar uses to write from. The very process of gathering data creates impressions, recollections, sensibilities, and a general feel for a person, place, or context all of which are not fully describable, or that may not be captured in fieldnotes. These are referred to as the “head notes” (Ottenberg, 1990) that accompany our fieldnotes. Efforts to saturate fieldnotes with all these details would likely make such notes less likely to be interpretable, because the mass of information would be overwhelming. Our own data, for example, includes tens of thousands of pages of transcripts, notes, and other research materials. Saturating these data with the headnotes of the team of ethnographers who collected them would make them largely inaccessible for interpretive analysis. This also does not recognize a fundamental epistemic approach of ethnographers, one that relies upon a recognition of positionality and reflexivity. Fieldnotes are not “objectively” constructed; they are positionally constituted, conditional on the relationships the subject-observer has with the research-subject.

In the second pathway, making parallel observations, Scholar B should be able to generate the same association between two variables as Scholar A by drawing an equivalent sample to Scholar A and then using their analytic strategy. This pathway is also unlikely to apply to ethnography because producing the same observations is unrealistic for two reasons. The first is, simply, time. New and unique observations will be generated in a different place/time from the original observations. Those two sets of observations are unlikely to align (though this is not necessarily evidence of a lack of reliability). Second, a different observer is likely to have different interactions with subjects. This post-positivist epistemic stance is an important distinction within the practice of ethnography. Both positionality and reflexivity suggest that parallel observations are unlikely to generate equivalent findings. As Murphy et al. (2021) note, “each ethnography is a snapshot of a vanished moment in time captured from the distinct perspective (or bias) of the researcher.”

Given these challenges, one might suggest that ethnography cannot be replicated and, therefore, it may not be a “science.” But the game has been fixed, grounded in assumptions not from ethnographic knowledge production, and instead from particular kinds of quantitative knowledge production, with its distinct epistemic culture. The very concept of replication is likely to be quite distinct within ethnographic inquiry, in part, because ethnographic work operates under a different logic of generalizability than that of most quantitative work. Ours is not a radical position; indeed, Freese and Peterson (2017) have noted that “replication is simply the wrong language to apply to qualitative studies” (159).

Let us be clear: quantitative epistemic culture is valuable and legitimate. It differs, however, from the equally valuable and legitimate ethnographic epistemic culture. The focus of ethnographic inquiry is not on empirical generalizability and replication. Some ethnographic work is highly descriptive, seeking to describe the world from the subject’s point of view, or provide an account of a group of people, a place, a kind of interaction, without any attempt to generalize. And when ethnographers do generalize, rarely do they seek to do so empirically. Instead, the ethnographic focus is on conceptual generalizability (Hirsch et al., 2010), which Collins et al. (2024) define as “Generalizations … not to a population of interest but instead to the abstracted concepts elucidated by observing an example or multiple examples of a case.”

Whereas quantitative sampling often rests on the assumption that (probability) sampled cases are generalizable to cases not sampled, ethnographic work operates under no such presupposition. In fact, we assume specific observations are unlikely to be reproduced. In part, this is because observations are temporally contingent and relationally bound to a particular observer. Patterns should be reproduceable, but specific empirical observations may not be. For example, in our own work on sexual violence (Hirsch and Khan, 2020), it is unlikely that we would have made the same specific observations had the institution we observed been a public university in a rural setting instead of an elite private one in the largest city in the United States. Or, if an observer were to “redo” our study at the same location just 3 years later, changes in student culture after COVID and shifts in the national conversation around sexuality would likely have had a considerable impact.

In this case, the critical feature for replication should not be whether specific observations were reproduced; it should be whether the conceptual framework applied or not. Does the conceptual apparatus continue to help make sense of the specific findings? This is a very different kind of logic of replication. And under such a logic, empirical reproducibility is not the standard. We wonder whether ethnography should be held to the broad conception of reanalysis as a “gold standard” in debates about open data as posed by Murphy et al. (2021). They define reanalysis as any attempt to “independently evaluate an ethnographer’s interpretations and consider alternative options” (2021, p. 44) and go on to present a set of different modes of evaluation, which include conducting secondary analyses of fieldnotes, re-visiting research sites, and comparing ethnographic interpretations to quantitative evidence. We argue that each of these strategies are hardly an appropriate means to the stated end. Their idealness is more reflective of the epistemic cultures driving open science than they are those of ethnographic practice. If embraced, they would likely construct a strong distinction between “scientific ethnography” (which would be viewed favorably) and ethnographic practice which does not take the epistemic culture of quantitative research as its ideal start point.

Secondary analysis does not question the facts but instead aims at evaluating the extent to which a body of data reasonably converges on a particular interpretation and subsequent theoretical implications. Given the sheer volume and intricacy of ethnographic data, not to mention the extent to which they are grounded in the researcher’s positionality and are marked by their partial perspective, we question the expedience of reanalysis for verification or replication on both a pragmatic and theoretical level. As for re-visiting research sites to see how a different researcher at a different time might reach different findings, comparing ethnographic findings to different forms of evidence, and testing for generalizability, all read more like mainstream scholarly practice which builds on former research to construct new research, rather than narrow replication/verification practices.

We are not arguing against the importance of reliability in ethnographic research. But the justification for open data rests on an epistemic foundation that differs from the epistemic foundations of ethnographic knowledge. Can the conceptual value be reproduced with other methods? Can a new set of observations generate not the same observations, but the same general patterns? In what ways do differently situated scholars generate different results because of their standpoint and because of how interactions change when the relational context between observer and observed also changes? Whereas the epistemological concerns within quantitative work can be solved by an open data approach (i.e., by specific observations reproduced), the epistemic foundations of qualitative work gives reproducibility a different meaning.

Put succinctly, reliability is important, but it is not “solved” by making data available, or by reproducing specific observations. This suggests that the response to the open data movement may look very different within ethnographic practice. Rather than start from the assumption that when it comes to addressing reliability, what is good for quantitative data (i.e., open data) should also be good for qualitative data, we should instead ask, “what is the qualitative problem around reliability, and how could it be better addressed?” The solutions to this question should be offered not on the basis of normative claims about desirability, but instead empirical study within our own community.

#### Political-economic/equity and efficiency

Most ethnography is simultaneously extremely cheap, and enormously costly. The reason for its trivial and exorbitant cost are the same: for the most part, ethnography involves one person (or in the case of our work, a small team of people) gathering information outside the time they must spend to sustain their life and career. The high cost is due to the absence of an economy of scale. On a community level, a lot of work is done that is underutilized. The low cost is attributed to graduate students and faculty for whom ethnography typically means conducting fieldwork between bouts of teaching. A lucky few have fellowships or sabbaticals, which exacerbate inequality within the field. This is in addition to other factors that result in significant inequalities within ethnographic production: familial/community commitments that interfere with the one’s ability to be in the field, lack of material resources required for moving to a specific location to gather data, and other resource constraints (e.g., time, money, etc.) required for other practicalities of research like transcription. These costs disproportionately affect more marginal scholars and they are different from the inequalities of, say, who receives privileged access to tax return data. The political-economic problems in ethnography are more about institutional challenges than they are about whether or not data are made accessible; qualitative scholars face problems with the support they receive (e.g., the logic of graduate programs, the organization of teaching, etc. are not structured with ethnographic research in mind). Scholars, especially marginal ones, require more support to do their work.

This is particularly important because focusing on the challenge of privileged access to data as the main political-economic problem may not result in a solution for more marginal scholars at all. While increased access to existing data may well reduce the cost of gathering information in general, more advantaged scholars will have determined what kinds of information are gathered. The data may provide a lot of information, but not the information a marginal researcher is interested in. For example, the recent American Voices Project (AVP) is an enormously valuable—and expensive—“qualitative census.” It asks a probability sample of Americans to tell their stories. The data provide a rich archive of life in the United States today. We have even used it ourselves (Caputo et al., forthcoming). But the data do not, for example, ask subjects about sexual identity. Researchers interested in queer life will find that AVP does not apply to their research interests. Using it is not an option; they’d need to gather their own data.

Providing access to data for more marginal researchers only addresses inequities if that data includes inquiries into the topics more marginal researchers are interested in. Large scale shared datasets are, by their nature, aimed at the core interest area of the discipline. Therefore, they are unlikely to generate much value to those who undertake research in more marginal areas of the discipline that would reflect their perspectives. Equity is an important scientific value, and while it is more likely than not to be improved when data becomes more accessible to quantitative scholars, we continue to see how qualitative data gathering is different. Equity means supporting work of marginalized scholars, not giving them access to work that others have produced.

#### Fraudulent findings and ethnography

The third primary justification for open data is regulatory: preventing fraud. Fraud happens. Within biomedical and life sciences papers, studies suggest that most retractions (67.4%) were because of fraud, duplicate publication, or plagiarism (Fang et al., 2012). Retractions in science journals are rare, and there is evidence that a small number of scientists are responsible for a disproportionate number of them (Brainard, 2018). We should be cautious of this claim, however, as one retraction may precipitate discovery of additional cases by the same person which suggests those who are “undiscovered” have not yet triggered their own retraction cascades. Within the natural sciences themselves, researchers who have investigated the link between data sharing requirements and article retractions or corrections have found none (Berberi and Roche, 2022). This suggests that open data may not increase corrections nor have significant impact on fraud.

Within qualitative research there are some well-known and rehearsed accusations of fraud, but there is little concrete evidence. Some scholars have been highly skeptical and critical of ethnographic knowledge and suggest a high probability of fraud (Lubet, 2017, 2019). Regardless of the degree of accuracy in these critiques, there is certainly fraud within qualitative research and the lack of concrete evidence suggests it has largely gone undiscovered. This is troubling, particularly given findings that textual evidence from mixed-methods research could not be verified in about one out of five studies, even upon contacting authors of studies in question (Moravcsik, 2014). The question is not whether there is fraud, but instead whether open data would make it more discoverable.

Given the overall absence of discoveries of fraud alongside the near certainty of its existence, it is difficult to argue that open data would have a negative effect upon reducing fraud. But so far the limited empirical evidence suggests that open data does not significantly reduce fraud so it cannot be expected to be an effective treatment for this problem. As we have learned in other contexts, punishment of those who violate our shared normative commitments can help reduce such violations. But scholars of punishment also note that when preventing norm violations, punishment is a relatively weak tool (Kleiman, 2009) that comes with a host of negative sequalae for communities (Fagan and Meares, 2008).

### Negative impact on data quality?

So far, most of this reflection has been in response to the logic of open data. Before we conclude, it is important to shift perspectives and consider some of the unintended consequences open data may have for qualitative scholars. We use our own work (Khan et al., 2018; Hirsch et al., 2019; Hirsch and Khan, 2020) as a guide. The motivating question is, “what would happen to the quality of ethnographic data if it were required to be broadly accessible?” We suggest that there may be perverse implications to open data—a movement meant to increase data quality—wherein data quality may decline.

Our own research would not have been possible with open data practices. We can say this with some degree of certainty because most victims of sexual violence elect not to engage in processes wherein their experiences could be known or identifiable. Such a claim runs counter to what Mozersky et al. (2020) found in their work, where they interviewed 30 people who participated in sensitive qualitative studies to better understand their concerns about data sharing. These interview subjects had participated in a health or sensitive health behavior study, involving topics like substance abuse and/or sexual behavior. While their study provides important *retrospective* accounts of how people felt, it does not provide a *prospective* understanding of what interview subjects would have said, had they known their data would be shared.

One of our works, “‘I did not want to be ‘that’ Girl’” (Khan et al., 2018) reflects the sentiment of many survivors of sexual violence. The research subjects did not want to be known as the ‘girl’ who was raped. Time and again, people were willing to tell us their stories in part because we promised to hold their stories close, do them justice, and protect their identities. We heard stories not only from people who experienced harm, but from those who committed it. We cannot fully predict what would have happened if we had told research subjects that their narratives would become available to others, but we have reasonable grounds to speculate: fewer than 5% of assaults are reported, and a primary reason is the potential publicity of that reporting (Mellins et al., 2017).

If these accounts were sufficiently anonymized to protect the research subjects, so many details would have to be removed or disassociated from the narratives that the data would become unusable. The questions of reliability would be senseless because other scholars would not have sufficient information to reproduce or even evaluate our claims. What if a reader of our open data did not know that someone who told us about assaulting one of their classmates also told us about their own previous, extensive experiences with sexual harm? The story would be profoundly transformed; the explanations would likely be grounded in a lack of information and understanding. What about instances wherein we, as researchers, could compare accounts because we knew two of our interview subjects were dating? But linking them would threaten their privacy. De-linking this information would be necessary but would not give an external party to our research the capacity to fully understand or even evaluate our claims. While for quantitative work there are extremely reasonable and credible justifications for open data—including improved data quality—the general practice is to remove data that is highly identifiable (so, where the “cells” contain fewer than 5 observations). For ethnographic work, the “richness” of the data is conditional, in part, on producing “cells” of one. De-identifying people to the point where they shared enough in common with others to be undiscoverable would rending data useless for the purposes of not only replication, but for any analysis at all. For most ethnographic work data quality and usability would likely decline should information become more accessible. Stories would go untold. Key facts would remain missing. We would likely know less, not more.

Of course, ours is a special case. But it is not a rare one. Nearly 50% of women will experience an assault in their lifetime. And there are other kinds of experiences (e.g., family abuse, suicidality, transphobia, systemic racism, etc.) that subjects are unlikely to potentially make public because of their existing marginality and the risks their stories being revealed to others carry. This is to say nothing of those in particularly dangerous positions (e.g., LGBTQ identified people in nations where they risk death from revelation, political dissident groups in oppressive regimes, etc.). Open data can undermine the legitimacy of sensitive ethnographic studies; such studies often focus on the hardest problems that people face in the course of their lives.^2^ There are also the researchers themselves. In contexts where ethnographers study violence and crime, authors have argued that “unmasking” “can get an ethnographer harmed” (Contreras, 2019: 293). While the “obvious” solution may seem to be to allow special cases exemption from open data, we need to acknowledge that this would further undermine the legitimacy of research that is already hyper-scrutinized. Sexual assault findings are systemically questioned, deemed unbelievable, or viewed as ‘produced’ by motivated researchers. Return to the third sentence of this paragraph and ask, “did I want a citation here? Did I find it credible that this many women are sexually assaulted?” Decades of research shows this number to be quite stable and has been reproduced across contexts. What are we doing if we put an asterisk next to findings from fields that generate insights about harm to marginalized populations? An asterisk that indicates: “this work on more precarious people or on this sensitive subject is not as scientific as others, because it does not meet our standards of open data.”

We should also consider how making fieldnotes and other ethnographic documentation available impacts the documentation itself. Demands that we, as scholars, redact our fieldnotes to the point where they would be readily accessible to other scholars (thus preserving the anonymization promised to our subject), would profoundly harm the quality of the raw data. So much information would have to be lost by rule. This would be done for the unlikely chance that others might want to access it. We speculate that the harm to knowledge would be far more profound than the problem such a solution attempts to fix.

## Discussion/conclusion

In his classic and often quoted paper, “Whose Side Are We On,” Becker (1967) acknowledges the challenges of ethnographic science and suggests a path forward.

We can, I think, satisfy the demands of our science by always making clear the limits of what we have studied, marking the boundaries beyond which our findings cannot be safely applied… for instance, that we have studied the prison through the eyes of the inmates and not through the eyes of the guards and other involved parties. We warn people, thus, that our study tells us only how things look from a vantage point… (247)

We see the enduring wisdom in Becker. This is not to say that in embracing Becker’s perspective, ethnographic practice should ignore demands for data transparency in the information age. In fact, there have been important contributions to the exploration of how ethnographic practice should respond to these demands (Murphy et al., 2021). And as Freese et al. (2022) note, data can be transparent or reproducible without necessarily becoming “open.” Secure data repositories with clear policies and procedures for data security are one such example (see also, Pool, 2017).

Ricœur’s (1970) distinction between a “hermeneutics of suspicion” and a “hermeneutics of faith” is helpful in thinking through the interpretation of qualitative data. Advocates of a hermeneutics intent on restoring meaning offer the concept of faith as a corrective to the hegemony of interpretative practices that are founded in suspicion. Ricœur famously named Freud, Marx, and Nietzsche fathers of suspicion, in that they offer models of symptomatic interpretation, viewing phenomena as they present themselves as surface-level artifacts of a deeper pattern prescribed by their strong theories.

We wish to broaden the application of this distinction beyond the realm of data to the interpretation of scholarship. Given the powerful tools at the disposal of quantitative social scientists who readily acknowledge their sensitivity to specifications, the relative transparency of quantitative data, and the often performatively “scientistic” nature of their claims, we accept that a somewhat high level of suspicion might be necessary when evaluating causal work. This, in turn, serves as solid ground for a legitimate application of an open data framework to quantitative social science.

On the other hand, given the intrinsically interpretative and narrative nature of much ethnographic work, we cautiously offer a more restorative approach to its evaluation. To best read ethnography, we must, to some extent, suspend our suspicion. To be sure, we are in no way advocating for a non-critical engagement with ethnographic work, but a strongly suspicious approach to the facts as they are presented seems impractical to us. This is in part because ethnographic replication is not about the facts being reproducible; it’s about the interpretive capacity of the conceptual framework produced from those facts. Importantly, as an epistemic community we address suspicions through more work on a topic—as a community of scholars that consistently re-engages arguments to evaluate their quality and capacity for explanation.

Put simply, if someone does not want to believe the ethnographer or if the tools at the ethnographer’s disposal seem inherently unscientific to any given reader, nothing the ethnographer does will be enough to appease that suspicion – open data practices included. We are therefore skeptical of applying open data practices to ethnography, not least out of a concern that the process of introducing broader open science logics might play into the delegitimization of interpretative research.

Our skeptical assessment of the open data movement and its impact on ethnography may be read as too definitive. There are three implications to our claims. First, we have suggested that the three primary justifications for open data—epistemic, political-economic, and regulatory—may not fully apply to the ethnographic context. Second, we have asked whether the presumed improvements to data quality may instead have negative impacts. And finally, even if we are wrong regarding both claims, we have asked whether the subsequent data would even be useful. What would it mean to have a dataset without a codebook?

We advise caution before requiring ethnographers to comply with open data requirements. Just as qualitative researchers do not import epistemic and ontological understandings from more quantitative work, so too should they not unquestioningly import the reasoning, justification, and solutions provided by the broader open science framework. Instead, we end with two suggestions. First, the inquiry into open science be scientific and not ideological, with support for research on the impacts of different solutions rather a simple commitment to a particular movement. It is not unique for a scientific movement to rest primarily on normative claims rather than evidence, but there is some irony to the relative lack of empirical work supporting open science, and open data in particular. Ethnographers have an opportunity to begin from a different place: that of study. Second, instead of importing the problem, justifications, and solutions from our natural science colleagues, we should instead look to our own challenges so we can fashion solutions that improve our understanding of the social world.

## Author contributions

SK: Writing – original draft, Writing – review & editing. JH: Writing – review & editing. OZ-Z: Writing – original draft, Writing – review & editing.

## Footnotes

^1^We have strongly advocated for instrument sharing, which can allow for a kind of replication (Hirsch et al., 2010; Hirsch and Khan, 2020); in this piece we are principally concerned, however, with data accessibility.

^2^We would like to thank the third reviewer of this paper for this sentence.
